# Comparison of clinical outcomes between ticagrelor and clopidogrel in patients with acute coronary syndrome and left ventricle dysfunction undergoing percutaneous coronary intervention: An observational study

**DOI:** 10.1097/MD.0000000000039620

**Published:** 2024-09-06

**Authors:** Caitong Zhao, Changdong Fei, Renzheng Chen, Yupeng Liu, Hualin Zhang

**Affiliations:** a Department of Quality Control, the General Hospital of Northern Theater Command, Shenyang, China; b Department of Health Management Center, the 967th Hospital of Joint Logistics Support Force of Chinese PLA, Dalian, China; c National Clinical Research Center of Geriatric Diseases, the Second Medical Center, Chinese PLA General Hospital, Beijing, China; d Department of Critical Care Medicine, the 967th Hospital of Joint Logistics Support Force of Chinese PLA, Dalian, China; e Department of Emergency, the 967th Hospital of Joint Logistics Support Force of Chinese PLA, Dalian, China.

**Keywords:** acute coronary syndrome, clinical outcome, dual antiplatelet, left ventricular dysfunction

## Abstract

Patients with acute coronary syndrome (ACS) and left ventricular (LV) dysfunction undergoing percutaneous coronary intervention (PCI) need adequate antithrombotic protection. We aim to compare the clinical outcomes between ticagrelor and clopidogrel in these patients. In total, 336 patients with ACS and LV dysfunction who undergoing PCI were included in this retrospective observational study. Of these, 137 received clopidogrel and 199 received ticagrelor. There was a 6-month follow-up period during which clinical outcomes were monitored. The incidence of the composite endpoint (23.1% vs 13.9%, *P* = .041) and bleeding events (6.5% vs 1.5%, *P* = .027) in the ticagrelor group were significantly higher compared to the clopidogrel group. Multivariate logistic regression analysis revealed that age (*P* = .006), hypertension (*P* = .007), liver insufficiency (*P* = .022), previous MI (*P* = .014) and ticagrelor (*P* = .044) were independent risk factors that affect the efficacy outcome. Age (*P* = .027) and ticagrelor (*P* = .016) were the independent risk factors for the safety outcome. Furthermore, in Cox survival regression analysis model, the survival rate of the efficacy endpoint in the clopidogrel group was seemingly higher than in the ticagrelor group (HR = 1.68, 95% CI: 0.97–2.90, *P* = .065). The survival rate of the bleeding endpoint in the clopidogrel group was higher than in the ticagrelor group (HR = 2.00, 95% CI: 1.17–3.40, *P* = .011). Compared to clopidogrel, ticagrelor showed increased risk of efficacy outcome and major bleeding events during 6-month follow-up in patients with ACS and LV dysfunction undergoing PCI.

## 1. Introduction

Acute coronary syndrome (ACS) is a severe manifestation of coronary atherosclerotic heart disease, characterized by its acute nature and often associated with a significantly increased risk of mortality.^[1]^ Currently, with the continuous improvement of percutaneous coronary intervention (PCI) therapy and the optimization of antithrombotic strategy, coupled with the improvement of medical conditions, the survival prognosis of ACS patients have been improved to a certain extent.^[2,3]^ However, the presence of specific comorbidities, such as anemia and diabetes, can further worsen the negative impact of clinical outcomes.^[4,5]^ Due to limited tolerance to hemodynamic stressors and ischemic complications, patients with left ventricular (LV) dysfunction who have undergone PCI are at an increased risk of developing adverse events,^[6,7]^ this highlights the need of adequate antithrombotic protection for these patients.

Dual antiplatelet therapy (DAPT), consisting of aspirin and a P2Y12 receptor inhibitor, is widely considered the standard treatment for patients with ACS.^[8]^ However, these patients are more likely to experience a major bleeding event in the first year of treatment with established DAPT,^[9]^ so this considerable risk offsets the clinical benefit of reducing the occurrence of ischemia. As a new oral P2Y12 inhibitor, ticagrelor exhibits faster and stronger antiplatelet aggregation properties compared to clopidogrel.^[10]^ Despite the controversy, ticagrelor has been demonstrated as the preferred treatment option in patients with ACS undergoing PCI without increasing the overall risk of major bleeding, especially for those who have simultaneous organ comorbidities.^[11]^ Nevertheless, it is imperative to thoroughly reassess the efficacy and safety of ticagrelor in East Asian populations, as genetic differences have been shown to result in reduced responsiveness to antiplatelet therapy.^[12]^

There is no convincing evidence that the presence of LV dysfunction could affect selection of antiplatelet agents. Additionally, there is a lack of available information concerning about the clinical impact of ticagrelor compared with clopidogrel in East Asian patients with ACS and LV dysfunction undergoing PCI. Thus, we designed this retrospective study to compare the clinical outcomes between ticagrelor and clopidogrel in patients with ACS and LV dysfunction who underwent PCI over a 6-month period, and hopefully provide contribute data in determining the optimal regimen.

## 2. Material and methods

### 2.1. Study population and ethical considerations

This was a single-center retrospective cohort study and we enrolled 336 patients with ACS and LV dysfunction who underwent PCI in the 967th Hospital of Joint Logistics Support Force of Chinese PLA between 2019 and 2023. ACS is defined based on a diagnosis of unstable angina pectoris or acute myocardial infarction (MI). Unstable angina is defined as the presence of myocardial ischemia without elevated troponin, with or without ECG ischemic changes. Acute MI encompasses both ST elevation MI and non-ST elevation MI. LV dysfunction was characterized by low absolute values (<50%) or significant decreases (20% relative to baseline) in cardiac LV ejection fraction (EF) before performed PCI.^[13]^ Exclusion criteria included contraindications to antiplatelet drugs, need for oral anticoagulant therapy, concomitant chronic infections, malignancies, and autoimmune diseases. Additionally, cases in which information was lost during the follow-up period were also excluded. Informed consent was obtained from each patient and the study protocol conforms to the ethical guidelines of the 1975 Declaration of Helsinki as reflected in a priori approval by the Human Ethics Committee of the 967th Hospital of Joint Logistics Support Force of Chinese PLA (No. PLA967-GC2023-120).

### 2.2. Clinical protocol

All patients underwent PCI using a conventional approach and were administered antiplatelet medications including aspirin and clopidogrel or ticagrelor. Patients were divided into 2 groups according to antiplatelet treatment. Patients in the ticagrelor group received a loading dose of 180 mg, followed by oral ticagrelor at a dose of 90 mg, taken twice daily, while patients in the clopidogrel group received a loading dose of 300 mg, followed by a maintenance dosage of 75 mg per day. Throughout the entire duration of the study, all patients received oral aspirin at a dosage of 100 mg once daily.

### 2.3. Data collection

All the data were extracted from electronic and traditional clinical medical records, including the patients’ demographic information, comorbidities, nursing records, clinical diagnosis, medications at the time of admission and discharge, interventions or procedures, and in-hospital biochemistry tests and echocardiography results. Two staffs with specialized training meticulously examined all of the data. A third researcher adjudicated any differences in interpretation between the 2 workers.

### 2.4. Follow-up and clinical outcomes

**Figure 1.**
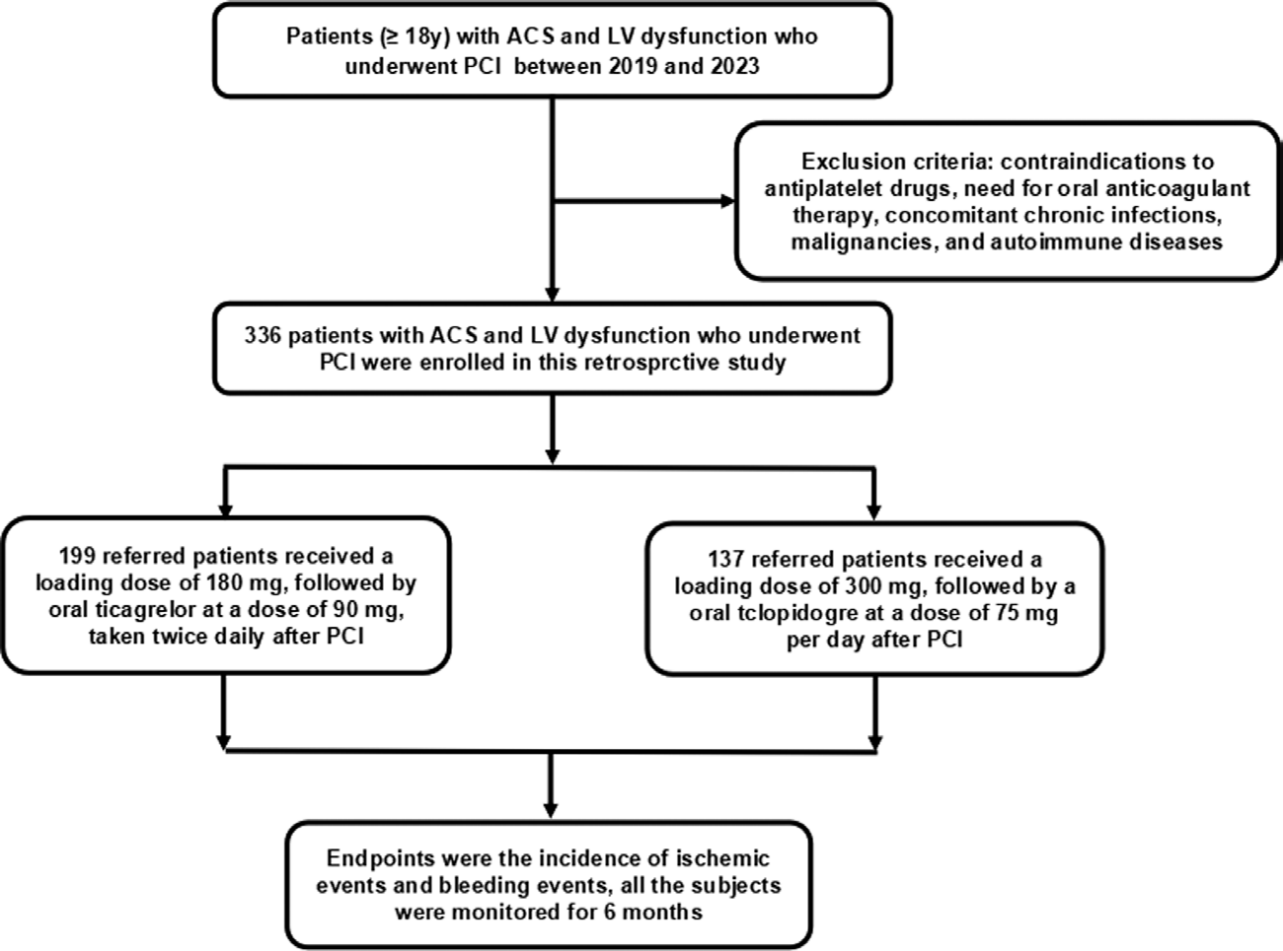
Flowchart of the study. ACS = acute coronary syndrome, LV = left ventricular, PCI = percutaneous coronary intervention.

The endpoints were the incidence of ischemic events (death, nonfatal MI, stroke, target vessel revascularization, and rehospitalization) and bleeding events.^[14]^ MI was defined based on the established clinical practice.^[15]^ Target vessel revascularization was defined as percutaneous revascularization or bypass surgery that specifically addressed the target lesion. Rehospitalization was defined as hospitalization for the treatment of unstable angina. Ischemic stroke was defined as neurological dysfunction caused by a focal infarction of the brain, spinal cord, or retina. The Bleeding Academic Research Consortium (BARC) standards were used to assess bleeding events.^[16]^ BARC bleeding types range from 0 (no bleeding) to 5 (fatal bleeding) and bleeding events that are classified as BARC 1 through 5. The clinical outcomes were monitored for 6 months underwent PCI across various groups (Fig. 1).

### 2.5. Statistical analysis

Continuous variables are reported as median (interquartile range, IQR), while categorical variables are expressed as n (%). Differences in measurements between different groups with normal distribution were tested using an independent-sample *t*-test, while the Mann–Whitney *U* test was used to analyze the data that did not fit a normal distribution. Categorical data were compared using the chi-square test, continuity correction, or Fisher exact test, as appropriate. Binary logistic regression model was employed to ascertain the independent predictors. Multivariable Cox-proportional hazard regression model (Enter/LR forward) was used for survival analyses. Variables that were considered clinically relevant or showed a univariate relationship with the outcomes (*P* < .10) were included in the multivariate regression model. To ensure the parsimony of the final model, variables for inclusion were carefully selected according to the number of events available and avoid the collinearity. Odds ratio (OR) or hazard ratio (HR) was employed in regression model to determine the relationship between risk factors and outcomes. *P* < .05 was considered statistically significant. Statistical analyses were performed using the SPSS software version 27 (IBM Corp., Armonk, NY, USA).

## 3. Results

### 3.1. Baseline characteristics

A total of 336 ACS patients with LV dysfunction were enrolled in this study between 2018 and 2022. A 6-month follow-up period concluded in December 2022. The patients were divided into the ticagrelor group (n = 199) and the clopidogrel group (n = 137) according to medicine treatment. Baseline characteristics of the 2 treatment groups were listed in Table 1. The size of LV diameter in the ticagrelor group were larger compared to the clopidogrel group (*P* = .048). And we observed a higher prevalence of chronic kidney disease among patients treated with ticagrelor than those treated with clopidogrel (*P* = .031).

**Table 1:** Baseline characteristics in ACS patients with LV dysfunction undergoing PCI.

	Total (n = 336)	Ticagrelor plus aspirin (n = 199)	Clopidogrel plus aspirin (n = 137)	*P* value
Age, years	62.00 (54.00–67.00)	62.00 (54.00–67.00)	63.00 (56.00–68.00)	.245
Males, n (%)	228 (67.9%)	141 (70.9%)	87 (63.5%)	.156
BMI, kg/m^2^	24.50 (22.49–26.89)	24.51 (22.13–27.18)	24.61 (22.62–26.70)	.660
Current smoker, n (%)	172 (51.2%)	103 (51.8%)	69 (50.4%)	.802
Current drinker, n (%)	148 (44.0%)	91 (45.7%)	57 (41.6%)	.454
UAP, n (%)	256 (76.2%)	155 (77.9%)	101 (73.7%)	.378
STEMI, n (%)	41 (12.2%)	21 (10.6%)	20 (14.6%)	.266
NSTEMI, n (%)	39 (11.6%)	23 (11.6%)	16 (11.7%)	.973
Heart rate, bpm	78.00 (70.00–87.00)	79.00 (70.00–88.00)	77.00 (68.00–85.50)	.189
SBP, mm Hg	131.50 (117.00–142.00)	130.00 (116.00–142.00)	131.00 (118.00–141.00)	.996
DBP, mm Hg	73.00 (64.25–82.00)	74.00 (63.00–82.00)	73.00 (65.50–81.50)	.892
Left ventricular diameter, mm	51.00 (48.00–55.00)	52.00 (48.00–56.00)	51.00 (48.00–55.00)	.048
Ejection fraction, n (%)	45.00 (42.00–48.00)	45.00 (42.00–48.00)	45.00 (43.00–48.00)	.636
History				
Atherosclerotic peripheral vascular disease, n (%)	118 (35.2%)	69 (34.7%)	49 (35.8%)	.837
Diabete, n (%)	87 (25.9%)	53 (26.6%)	34 (24.8%)	.709
Previous MI, n (%)	57 (17.0%)	35 (17.6%)	22 (16.1%)	.714
Previous coronary stent implantation, n (%)	58 (17.3%)	36 (18.1%)	22 (16.1%)	.628
Previous GI bleeding, n (%)	12 (3.6%)	7 (3.5%)	5 (3.6%)	.949
Hypertension, n (%)	225 (67.0%)	128 (64.3%)	97 (70.8%)	.215
Hyperuricemia, n (%)	21 (6.3%)	12 (6.0%)	9 (6.6%)	.841
Hyperlipemia, n (%)	80 (23.8%)	44 (22.1%)	36 (26.3%)	.378
Liver insufficiency, n (%)	21 (6.3%)	15 (7.5%)	6 (4.4%)	.240
Chronic kidney disease, n (%)	40 (11.9%)	30 (15.1%)	10 (7.3%)	.031
Ischemic stroke, n (%)	40 (11.9%)	23 (11.6%)	17 (12.4%)	.813
Medication				
Statins, n (%)	325 (96.7%)	192 (96.5%)	133 (97.1%)	.762
Nitrate, n (%)	66 (19.6%)	32 (16.1%)	34 (24.8%)	.723
Beta blockers, n (%)	255 (75.9%)	154 (77.4%)	101 (73.7%)	.440
RAAS inhibitors, n (%)	270 (80.4%)	160 (80.4%)	110 (80.3%)	.980
Calcium channel blockers, n (%)	71 (21.1%)	33 (16.6%)	38 (27.7%)	.443
Proton pump inhibitors, n (%)	175 (52.1%)	102 (51.3%)	73 (53.3%)	.715
Insulin, n (%)	59 (17.6%)	37 (18.6%)	22 (16.1%)	.548
Other hypoglycemic agents, n (%)	35 (10.4%)	19 (9.5%)	16 (11.7%)	.530
Biomedical indicators				
Leucocyte, 10^9^/L	6.94 (5.90–8.28)	6.69 (5.90–8.30)	6.79 (5.95–8.00)	.491
Neutrophil percentage, n(%)	64.25 (58.33–70.88)	64.90 (58.40–71.30)	63.30 (58.10–69.95)	.337
Hemoglobin, g/L	131.50 (120.00–144.00)	133.00 (119.00–144.00)	130.00 (120.00–142.00)	.370
Platelets, 10^9^/L	189.00 (151.00–222.00)	189.00 (154.00–224.00)	190.00 (147.00–218.50)	.755
Mean platelet volume, fL	11.40 (10.00–12.50)	11.30 (9.90–12.30)	11.60 (10.30–12.60)	.087
Platelet distribution width, fL	16.20 (14.30–17.10)	16.10 (14.50–17.00)	16.20 (14.20–17.30)	.421
ALT, U/L	24.30 (17.40–36.30)	24.80 (17.70–36.30)	23.70 (17.00–36.40)	.615
AST, U/L	21.40 (16.30–29.78)	22.20 (16.80–32.40)	21.20 (15.70–27.80)	.103
Creatinine, μmol/L	77.10 (63.20-.92.13)	78.30 (64.90–94.70)	74.50 (61.55–89.15)	.184
eGFR, mL/min	88.0 (73.00–96.00)	87.00 (74.00–95.00)	88.00 (72.50–97.00)	.787
Total cholesterol, mmol/L	3.76 (3.11–4.49)	3.76 (3.10–4.53)	3.78 (3.12–4.48)	.749
Triglyceride, mmol/L	1.47 (1.13–1.96)	1.47 (1.09–1.92)	1.47 (1.19–2.12)	.482
Myoglobin,	33.90 (33.90–68.53)	49.50 (35.60–71.70)	44.80 (31.75–63.75)	.124
Hs-cTnI, ng/mL	0.05 (0.02–0.05)	0.05 (0.02–0.05)	0.05 (0.02–0.05)	.744
Glycosylated hemoglobin, %	6.35 (5.30–7.40)	6.40 (5.40–7.50)	6.30 (5.30–7.30)	.390
FBG, mmol/L	5.67 (4.56–7.15)	5.56 (4.50–6.81)	5.98 (4.72–7.32)	.141
Coronary angiography				
Single-vessel disease, n (%)	89 (26.5%)	60 (30.2%)	29 (21.2%)	.067
Double-vessel disease, n (%)	127 (37.8%)	71 (35.7%)	56 (40.8%)	.334
Triple-vessel disease, n (%)	120 (35.7%)	68 (34.4%)	52 (38.0%)	.477

Data were expressed as n (%) and median (IQR); IQR, interquartile range; *P* value, Mann–Whitney *U* test or Pearson Chi-square test.

ACS = acute coronary syndrome, ALT = alanine aminotransferase, AST = aspartate aminotransferase, BMI = body mass index, DBP = diastolic blood pressure, eGFR = estimated glomerular filtration rate, FBG = fasting blood glucose, GI = gastrointestinal, Hs-cTnI = hypersensitive cardiac troponin I, LV = left ventricular, MI = myocardial infarction, NSTEMI = non-ST-segment elevation myocardial infarction, PCI = percutaneous coronary intervention, RAAS = renin-angiotensin-aldosterone system, SBP = systolic blood pressure, STEMI = ST-segment elevation myocardial infarction, UAP = unstable angina pectoris.

### 3.2. Clinical outcomes

At 6 months, the proportion of the composite endpoint in the clopidogrel group was lower than in the ticagrelor group in terms of efficacy outcome, but there were no significant differences of the related ischemic subfactors between the 2 groups (23.1% vs 13.9%, *P* = .041). In the safety outcome, there were more bleeding events defined with BARC in the ticagrelor group compared to the clopidogrel group (25.6% vs 13.9%, *P* = .009), especially in the BARC type 2 (6.5% vs 1.5%, *P* = .027). The bleeding risk in the ticagrelor group had a tendency to increase (Table 2).

**Table 2:** Clinical outcomes in ACS patients with LV dysfunction undergoing PCI.

	Total (n = 336)	Ticagrelor plus aspirin (n = 199)	Clopidogrel plus aspirin (n = 137)	*P* value
Efficacy outcome				
Composite endpoint	65 (19.3%)	46 (23.1%)	19 (13.9%)	.041
Death	8 (2.4%)	6 (2.5%)	2 (1.5%)	.579
MI	1 (0.3%)	1 (0.5%)	0 (0.0%)	1.000
Stroke	5 (1.5%)	3 (1.5%)	2 (1.5%)	1.000
Revascularization	26 (7.7%)	17 (8.5%)	9 (6.6%)	.506
Rehospitalization	25 (7.4%)	19 (9.5%)	6 (4.4%)	.076
Safety outcome				
BARC 1 to 5	70 (20.8%)	51 (25.6%)	19 (13.9%)	.009
BARC 1	46 (13.7%)	31 (15.6%)	15 (10.9%)	.225
BARC 2	15 (4.5%)	13 (6.5%)	2 (1.5%)	.027
BARC 3	6 (1.8%)	4 (2.0%)	2 (1.5%)	1.000
BARC 4	1 (0.3%)	1 (0.5%)	0 (0.0%)	1.000
BARC 5	2 (0.5%)	2 (1.0%)	0 (0.0%)	.513

Data were expressed as n (%) and median (IQR); IQR, interquartile range; *P* value, Pearson Chi-Square test, Continuity correction test or Fisher exact test.

Composite endpoint including: death, nonfatal MI, stroke, target vessel revascularization, and rehospitalization.

ACS = acute coronary syndrome, BARC = Bleeding Academic Research Consortium definition for bleeding, LV = left ventricular, MI = myocardial infarction, PCI = percutaneous coronary intervention.

### 3.3. Risk factors

Demographic characteristics, medical history, medications, cardiac function, biomedical indicators, results of coronary angiography and drug use were included in the logistic regression model analysis (Tables S1 and S2, Supplemental Digital Content, http://links.lww.com/MD/N520). After calibration analysis, multivariate model showed that age (*P* = .006), hypertension (*P* = .007), liver insufficiency (*P* = .022), previous MI (*P* = .014) and ticagrelor (*P* = .044) were independent risk factors that affected the efficacy outcome (Table 3). The same logistic regression model was conducted, and age (*P* = .027) and ticagrelor (*P* = .016) were independent risk factors for the safety endpoint (Table 4).

**Table 3:** Risk factors for composite endpoint of ACS patients with LV dysfunction undergoing PCI in multivariable analysis.

Variable	Multivariable OR (95% CI)	P1 value	Multivariable HR (95% CI)	P2 value
Age, yr	1.06 (1.02–1.10)	.006	1.03 (1.00–1.07)	.040
Ejection fraction	–	–	–	–
History				
Hypertension	2.65 (1.31–5.35)	.007	2.13 (1.23–4.04)	.020
Liver insufficiency	3.29 (1.19–9.08)	.022	2.78 (1.32–5.85)	.007
Chronic kidney disease	–	–	1.98 (1.07–3.66)	.031
Previous MI	2.38 (1.19–4.75)	.014	2.28 (1.32–3.94)	.003
Biomedical indicator				
Hemoglobin	–	–	–	–
eGFR	–	–	–	–
Grouping				
Ticagrelor vs clopidogrel (as reference)	1.88 (1.02–3.48)	.044	–	–

95% CI = 95% confidence interval, ACS = acute coronary syndrome, eGFR = estimated glomerular filtration rate, HR = hazard ratio, LV = left ventricular, MI = myocardial infarction, OR = odds ratio, P1 = Logistic regression analysis, P2 = COX survival analysis, PCI = percutaneous coronary intervention.

**Table 4:** Risk factors for BARC of ACS patients with LV dysfunction undergoing PCI in multivariable analysis.

Variable	Multivariable OR (95% CI)	P1 value	Multivariable HR (95% CI)	P2 value
Age, years	1.04 (1.00–1.08)	.027	1.04 (1.01–1.07)	.018
**History**				
Chronic kidney disease	2.05 (0.97–4.34)	.061	1.97 (1.09–3.56)	.025
Previous coronary stent implantation	1.93 (1.00–3.76)	.052	1.59 (0.92–2.73)	.098
Previous GI bleeding	2.19 (0.58–8.29)	.249	1.74 (0.64–4.74)	.276
Biomedical indicator				
Triglyceride	1.20 (0.94–1.53)	.140	1.11 (0.98–1.27)	.107
Glycosylated hemoglobin	1.20 (0.99–1.39)	.073	1.15 (0.99–1.33)	.062
Grouping				
ticagrelor vs clopidogrel (as reference)	2.08 (1.15–3.85)	.016	2.00 (1.17–3.40)	.011

95% CI = 95% confidence interval, ACS = acute coronary syndrome, BARC = Bleeding Academic Research Consortium definition for bleeding, eGFR = estimated glomerular filtration rate, GI = gastrointestinal, HR = hazard ratio, LV = left ventricular, OR = odds ratio, P1 = Logistic regression analysis, P2 = COX survival analysis, PCI = percutaneous coronary intervention.

Univariate and multivariate Cox-proportional hazard regression models were employed to identify the risk factors influencing the clinical outcomes (Tables S3 and S4, Supplemental Digital Content, http://links.lww.com/MD/N520), and the inclusion variables were as mentioned above. After calibration analysis, age (*P* = .040), hypertension (*P* = .020), liver insufficiency (*P* = .007), chronic kidney disease (*P* = .031) and previous MI (*P* = .003) were found to be independent factors influencing the survival rate of the efficacy endpoint in the multivariate model (Table 3). Moreover, age (*P* = .018) and chronic kidney disease (*P* = .025) were found to be independent factors influencing the survival rate of the safety endpoint in the multivariate model (Table 4).

### 3.4. Survival analysis

**Figure 2.**
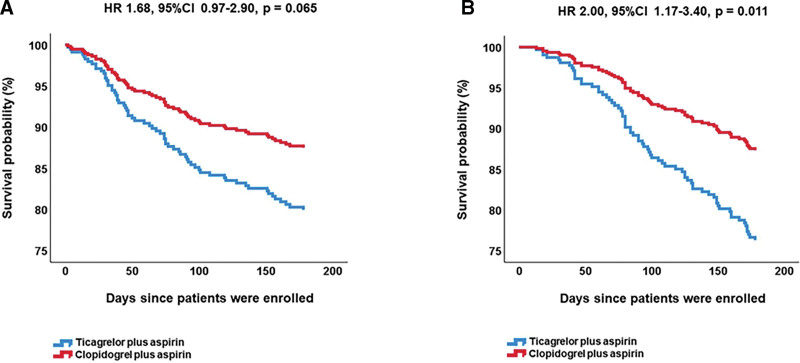
(A) Event-free survival for composite of efficacy outcome in ACS patients with LV dysfunction. The survival rate of the efficacy endpoint in the clopidogrel group did not exhibit significant difference compared to in the ticagrelor group. (B) Event-free survival for bleeding events defined by the BARC criteria in ACS patients with LV dysfunction. The survival rate of the bleeding endpoint in the clopidogrel group was higher than in the ticagrelor group.

We further compared the endpoint events in the 6-month follow-up between the 2 different treatment groups. We observed that the survival rate of the composite endpoint in the clopidogrel group was seemingly higher than in the ticagrelor group, although this difference did not reach statistical significance (HR = 1.68, 95% CI: 0.97–2.90, *P* = .065) (Fig. 2A). The survival rate of the bleeding endpoint in the clopidogrel group was higher than in the ticagrelor group (HR = 2.00, 95% CI: 1.17–3.40, *P* = .011) (Fig. 2B).

## 4. Discussion

To our knowledge, few studies focused on the comparative effectiveness and safety of various antiplatelet regimens in patients with ACS and LV dysfunction undergoing PCI. The main findings of our study conducted on a Chinese population indicated that ticagrelor had a negative impact on the survival rate of the efficacy outcome and increased the all-cause occurrence of safety outcome in patients with ACS and LV dysfunction compared to clopidogrel.

LV dysfunction is a complex clinical syndrome characterized by abnormalities in ventricular systole and diastole, caused by abnormal cardiac function and structure. Patients with reduced LV EF demonstrated a higher burden of cardiovascular risks and comorbidities, along with more intricate coronary anatomy and lesional features.^[17]^ For patients with ACS and LV dysfunction, PCI is the preferred treatment which improving myocardial blood perfusion, activating hibernating cells in necrotic myocardium, and promoting cardiac remodeling and functional recovery.^[18]^ Compared to medical treatment alone, it offers superior clinical benefits.^[19]^ Previous studies have demonstrated that many risk factors weigh heavy for the clinical outcomes of patients after PCI.^[14,20–22]^ Gallone et.al^[13]^ discovered that decreased LV EF (<40%) served as a strong predictor for predicting long-term adverse clinical outcomes in patients who underwent PCI. Moreover, each 10% increase in LV EF being associated with a 44% reduction in 4-year post-PCI mortality.^[23]^ Besides, it has been observed that hemodynamic abnormalities and thrombotic events are more common in patients with LV dysfunction,^[24]^ and a sufficient antiplatelet effect in ACS patients would suppress micro thromboembolism induced by coronary reperfusion.^[25]^ Therefore, the potency of the antiplatelet regimen holds significant importance for patients with ACS and LV dysfunction.

Ticagrelor is developed as a novel P2Y12 receptor antagonist that remains unaffected by genetic polymorphism in CYP2C19. It circumvents the clinical limitations of clopidogrel, including liver metabolism, drug interactions, and gene polymorphisms that code for platelet receptors, resulting in more potent and predictable antiplatelet effects.^[26,27]^ Ticagrelor has been shown to enhance adenosine levels by inhibiting its reuptake by erythrocytes, and adenosine has the potential to regulate inflammatory response and promote vasodilation.^[28]^ The Global Phase III PLATO trial demonstrated that ticagrelor exhibited a significant reduction in the occurrence of major adverse cardiovascular events (MACEs) when compared to clopidogrel over a 12-month period. However, it was observed that ticagrelor carried a similar risk for major bleeding as clopidogrel.^[26]^ Another retrospective multicenter study showed that ticagrelor exhibited a superior benefit-risk profile for MACEs than clopidogrel in ACS patients.^[11]^ However, several studies have yielded divergent findings. Spoendlin et.al demonstrated that there was no significant difference observed in 2 separate comparisons between the ticagrelor and clopidogrel groups among patients with ACS.^[29]^ Similar results were also found in researches conducted in East Asian population with different ethnic groups.^[30–32]^ It is noteworthy that the ticagrelor group exhibited a significantly higher incidence of adverse cardiovascular outcomes compared to the clopidogrel group in our study. Based on the current clinical evidence, it can be inferred that ticagrelor exhibits an antithrombotic efficacy that is at least comparable to, if not superior to, clopidogrel in the majority of ethnic groups. This disparity might primarily be attributed to the varying composite endpoints across different studies. A deeper analysis revealed that a higher proportion of patients hospitalized for angina occurred in the ticagrelor group, rather than the MACE-related cardiovascular events. Furthermore, the variance in follow-up time might also have influenced the final clinical outcomes.

Antithrombotic therapy plays a crucial role as a secondary prevention in ACS patients after PCI. But antithrombosis benefits were partially offset by an elevated risk of bleeding events, which was strongly associated with increased mortality.^[33]^ The phenomenon known as the “East Asian Paradox” suggests that East Asian patients are more susceptible to experiencing bleeding complications related to antithrombotic therapy than non-East Asian patients.^[34]^ Ethnic differences play a significant role in the development of atherosclerotic thrombosis, as evidenced by variations in coagulation, fibrinolysis, and inflammation markers.^[35]^ Additionally, it is widely recognized that Asian patients exhibit a lower BMI, and a higher prevalence of CYP2C19 dysfunction gene carriers.^[36,37]^ Therefore, it is necessary to consider the intricate balance between ischemia and bleeding complications and further optimize the antiplatelet strategy to improving patient outcomes. The East Asian Phase III PHILO trial demonstrated that ticagrelor, in comparison to clopidogrel, exhibited a higher frequency of bleeding events but did not significantly reduce the composite primary efficacy endpoint.^[30]^ A recent study conducted in Taiwan has revealed that ticagrelor was associated with higher MACEs and major bleeding risk within 12 months in patients with ACS and who were on dialysis than clopidogrel.^[38]^ Besides, the use of ticagrelor has not been shown to improve the efficacy outcome but significantly increased the number of major bleeding events compared with clopidogrel treatment in ACS patients with diabetes mellitus from other East Asian countries.^[14,31]^

In our study, we also found that age and chronic kidney disease were potential risk factors of the composite (safety) endpoint. Moreover, our results demonstrated that different antithrombotic regimens had a significant impact on bleeding events during follow-up in patients with ACS and LV dysfunction in the multivariate model for calibration analysis. Studies have compared the prognostic differences of different regimens in people with combined risk factors, including those aged > 70 years old, female and with a history of diabetes mellitus.^[39–42]^ While there were little data about the clinical impact of ticagrelor compared with clopidogrel in patients with LV dysfunction. A previous study did not support the use of triple antithrombotic therapy (the addition of oral anticoagulation to DAPT) as a strategy over conventional ticagrelor-based DAPT for patients with apical dysfunction after MI who were treated with PCI.^[43]^ It is suggested that the implementation of a robust antithrombotic regimen may not yield superior clinical outcomes in patients with cardiac dysfunction after PCI. Our study also found that compared with clopidogrel, ticagrelor with stronger antithrombotic effect did not show better clinical benefit in patients with LV dysfunction undergoing PCI due to more bleeding events. Clopidogrel based DAPT may be a safer alternative than ticagrelor. In future research endeavors, it is imperative to accord greater emphasis to these unique individuals who possess specific comorbidities. By accumulating a robust corpus of evidence-based medical data, we can devise more tailored and rational postoperative medication protocols for patients undergoing PCI.

There are still several limitations in the present study. First, although nearly 5 consecutive years of patients were enrolled, the number of patients included was relatively small. The conclusions need to be further confirmed in multicenter studies with large samples. Second, the use of the study drugs may have varied among different centers because of some factors such as the financial ability of patients and the clinical experience of physicians. Thirdly, we did not collect sufficient postoperative out-of-hospital information, which may have an impact on the clinical prognosis, such as lifestyle, medication and other factors. Lastly, due to differences in baseline clinical characteristics that may have influenced our results, we corrected for these differences by performing a multivariable analysis that included a wide range of variables. However, the findings of this retrospective study should be interpreted cautiously due to the lack of a high-standard match.

## 5. Conclusion

In conclusion, the results of the present analysis indicated that patients with ACS and LV dysfunction who undergoing PCI had higher risks of experiencing the composite endpoint (death, nonfatal MI, stroke, target vessel revascularization, and rehospitalization) and bleeding events during the 6-month follow-up when treated with ticagrelor compared to clopidogrel. The efficacy and safety of ticagrelor needs to be approached with caution and we considered that clopidogrel may be a more suitable option over ticagrelor for this particular population. Furthermore, large-scale, long-term, randomized trials should be conducted imperatively to verify these conclusions.

## Acknowledgments

We appreciated all the subjects who participated in the study.

## Author contributions

**Conceptualization:** Renzheng Chen, Hualin Zhang.

**Data curation:** Caitong Zhao, Changdong Fei, Renzheng Chen.

**Formal analysis:** Caitong Zhao, Changdong Fei, Renzheng Chen.

**Funding acquisition:** Yupeng Liu.

**Investigation:** Caitong Zhao, Changdong Fei.

**Methodology:** Caitong Zhao, Changdong Fei, Renzheng Chen.

**Project administration:** Yupeng Liu, Hualin Zhang.

**Resources:** Yupeng Liu, Hualin Zhang.

**Software:** Caitong Zhao, Renzheng Chen.

**Supervision:** Yupeng Liu, Hualin Zhang.

**Validation:** Yupeng Liu, Hualin Zhang.

**Visualization:** Yupeng Liu.

**Writing – original draft:** Renzheng Chen, Hualin Zhang.

**Writing – review & editing:** Changdong Fei, Yupeng Liu.

## Supplementary Material


