# Supplementary material for: Comparison of clinical outcomes between ticagrelor and clopidogrel in patients with acute coronary syndrome and left ventricle dysfunction undergoing percutaneous coronary intervention: An observational study
Source: Medicine (Baltimore). 2024 Sep 6;103(36):e39620. doi: 10.1097/MD.0000000000039620 (PMC11383269; doi:10.1097/MD.0000000000039620)
Supplement: Supplementary file 1 [file medi-103-e39620-s001.doc]

**Supplemental table 1. Risk factors for composite endpoint** **in ACS patients with LV dysfunction undergoing PCI.**

| **Variable** | **Univariable**  **OR (95% CI)** | **p value** | **Multivariable**  **OR (95% CI)** | **p value** |
| --- | --- | --- | --- | --- |
| Age, years | 1.04 (1.00-1.08) | 0.036 | 1.06 (1.02-1.10) | 0.006 |
| Gender (female vs male) | 1.01 (0.57-1.80) | 0.975 |  |  |
| BMI, kg/m^2^ | 0.95 (0.88-1.03) | 0.210 |  |  |
| Current smoking | 0.91 (0.53-1.56) | 0.725 |  |  |
| Current drinking | 1.40 (0.81-2.41) | 0.225 |  |  |
| Left ventricular diameter | 1.03 (0.99-1.07) | 0.120 |  |  |
| Ejection fraction | 0.94 (0.88-1.00) | 0.052 |  |  |
| **History** | | | | |
| Hypertension | 2.54 (1.30-5.00) | 0.007 | 2.65 (1.31-5.35) | 0.007 |
| Diabetes | 1.12 (0.61-2.06) | 0.712 |  |  |
| Liver insufficiency | 2.79 (1.10-7.03) | 0.030 | 3.29 (1.19-9.08) | 0.022 |
| Chronic kidney disease | 2.26 (1.09-4.67) | 0.028 |  |  |
| Previous MI | 2.78 (1.20-4.32) | 0.012 | 2.38 (1.19-4.75) | 0.014 |
| Previous coronary stent implantation | 1.11 (0.55-2.24) | 0.776 |  |  |
| Ischemic stroke | 1.70 (0.80-3.61) | 0.168 |  |  |
| Hyperlipemia | 0.68 (0.34-1.34) | 0.262 |  |  |
| Previous GI bleeding | 2.16 (0.63-7.39) | 0.222 |  |  |
| Hyperuricemia | 1.74 (0.65-4.66) | 0.274 |  |  |
| **Medication** |  |  |  |  |
| RAAS inhibitors | 1.94 (0.88-4.30) | 0.102 |  |  |
| Beta blockers | 0.79 (0.43-1.46) | 0.452 |  |  |
| Proton pump inhibitors | 1.38 (0.80-2.38) | 0.253 |  |  |
| Insulin | 1.55 (0.80-3.00) | 0.195 |  |  |
| Statins | 0.63 (0.16-2.44) | 0.109 |  |  |
| **Biomedical indicators** |  |  |  |  |
| Leucocyte | 1.08 (0.96-1.21) | 0.231 |  |  |
| Hemoglobin | 0.99 (0.98-1.00) | 0.048 |  |  |
| Platelets | 1.00 (0.99-1.00) | 0.149 |  |  |
| Mean platelet volume | 0.96 (0.82-1.13) | 0.628 |  |  |
| Platelet distribution width | 1.03 (0.93-1.13) | 0.596 |  |  |
| ALT | 1.01 (1.00-1.019) | 0.134 |  |  |
| eGFR | 0.98 (0.97-1.00) | 0.024 |  |  |
| Triglyceride | 0.89 (0.67-1.18) | 0.423 |  |  |
| Total cholesterol | 0.88 (0.68-1.13) | 0.311 |  |  |
| Hs-cTnI | 1.01 (0.96-1.07) | 0.661 |  |  |
| Glycosylated hemoglobin | 1.02 (0.86-1.21) | 0.808 |  |  |
| **Coronary arteriography** |  |  |  |  |
| Single-vessel disease | ref | 0.783 |  |  |
| Double-vessel disease | 0.81 (0.40-1.65) | 0.564 |  |  |
| Triple-vessel disease | 1.03 (0.55-1.92) | 0.926 |  |  |
| **Grouping** |  |  |  |  |
| ticagrelor vs clopidogrel (as reference) | 1.87 (1.04-3.36) | 0.037 | 1.88 (1.02-3.48) | 0.044 |

95% CI, 95% confidence interval; OR, odds ratio;

ACS, Acute coronary syndrome; ALT, Alanine aminotransferase; BMI, Body mass index; eGFR, Estimated glomerular filtration rate; GI, Gastrointestinal; Hs-cTnI: hypersensitive cardiac troponin I; LV, left ventricular; MI, Myocardial infarction; PCI, percutaneous coronary intervention; RAAS, Renin-angiotensin-aldosterone system.

**Supplemental table 2. Risk factors for BARC in ACS patients with LV dysfunction undergoing PCI.**

| **Variable** | **Univariable**  **OR (95% CI)** | **p value** | **Multivariable**  **OR (95% CI)** | **p value** |
| --- | --- | --- | --- | --- |
| Age, years | 1.04 (1.00-1.07) | 0.045 | 1.04 (1.00-1.08) | 0.027 |
| Gender (female vs male) | 0.96 (0.55-1.69) | 0.886 |  |  |
| BMI, kg/m^2^ | 1.00 (0.96-1.04) | 0.906 |  |  |
| Current smoking | 1.26 (0.74-2.14) | 0.395 |  |  |
| Current drinking | 0.75 (0.44-1.29) | 0.300 |  |  |
| Left ventricular diameter | 1.00 (0.98-1.04) | 0.799 |  |  |
| Ejection fraction | 0.99 (0.93-1.06) | 0.787 |  |  |
| **History** | | | | |
| Hypertension | 1.42 (0.79-2.55) | 0.240 |  |  |
| Diabetes | 0.99 (0.54-1.80) | 0.969 |  |  |
| Liver insufficiency | 2.00 (0.78-5.16) | 0.152 |  |  |
| Chronic kidney disease | 2.31 (1.13-4.70) | 0.021 | 2.05 (0.97-4.34) | 0.061 |
| Previous MI | 1.30 (0.66-2.54) | 0.448 |  |  |
| Previous coronary stent implantation | 1.96 (1.04-3.68) | 0.038 | 1.93 (1.00-3.76) | 0.052 |
| Ischemic stroke | 1.12 (0.51-2.47) | 0.782 |  |  |
| Hyperlipemia | 1.25 (0.69-2.28) | 0.462 |  |  |
| Previous GI bleeding | 2.85 (0.88-9.26) | 0.082 | 2.19 (0.58-8.29) | 0.249 |
| Hyperuricemia | 0.38 (0.09-1.68) | 0.203 |  |  |
| **Medication** |  |  |  |  |
| RAAS inhibitors | 0.78 (0.41-1.48) | 0.448 |  |  |
| Beta blockers | 0.82 (0.45-1.48) | 0.505 |  |  |
| Proton pump inhibitors | 1.50 (0.88-2.56) | 0.138 |  |  |
| Insulin | 1 09 (0.55-2.16) | 0.803 |  |  |
| Statins | 1.19 (0.25-5.64) | 0.826 |  |  |
| **Biomedical indicators** |  |  |  |  |
| Leucocyte | 1.01 (0.89-1.13) | 0.932 |  |  |
| Hemoglobin | 1.00 (0.98-1.01) | 0.376 |  |  |
| Platelets | 1.00 (1.00-1.01) | 0.741 |  |  |
| Mean platelet volume | 1.10 (0.94-1.28) | 0.223 |  |  |
| Platelet distribution width | 0.97 (0.88-1.07) | 0.559 |  |  |
| ALT | 1.00 (0.99-1.01) | 0.793 |  |  |
| eGFR | 1.01 (0.99-1.02) | 0.665 |  |  |
| Triglyceride | 1.23 (1.00-1.52) | 0.051 | 1.20 (0.94-1.53) | 0.140 |
| Total cholesterol | 0.96 (0.76-1.22) | 0.738 |  |  |
| Hs-cTnI | 1.02 (0.96-1.07) | 0.560 |  |  |
| Glycosylated hemoglobin | 1.20 (1.02-1.41) | 0.033 | 1.20 (0.99-1.39) | 0.073 |
| **Coronary arteriography** |  |  |  |  |
| Single-vessel disease | ref | 0.307 |  |  |
| Double-vessel disease | 0.71 (0.35-1.46) | 0.350 |  |  |
| Triple-vessel disease | 1.23 (0.68-2.23) | 0.503 |  |  |
| **Grouping** |  |  |  |  |
| ticagrelor vs clopidogrel (as reference) | 2.14 (1.20-3.82) | 0.010 | 2.08 (1.15-3.85) | 0.016 |

95% CI, 95% confidence interval; OR, odds ratio;

ACS, Acute coronary syndrome; ALT, Alanine aminotransferase; BMI, Body mass index; eGFR, Estimated glomerular filtration rate; GI, Gastrointestinal; Hs-cTnI: hypersensitive cardiac troponin I; LV, left ventricular; MI, Myocardial infarction; PCI, percutaneous coronary intervention; RAAS, Renin-angiotensin-aldosterone system.

**Supplemental table 3. Correlation between** **composite endpoint with different** **antiplatelet drug regimens in ACS patients with LV dysfunction undergoing PCI.**

| **Variable** | **Univariable**  **HR (95% CI)** | **p value** | **Multivariable**  **HR (95% CI)** | **p value** |
| --- | --- | --- | --- | --- |
| Age, years | 1.03 (1.00-1.07) | 0.042 | 1.03 (1.00-1.07) | 0.040 |
| Gender (female vs male) | 1.02 (0.61-1.71) | 0.946 |  |  |
| BMI, kg/m^2^ | 0.96 (0.89-1.03) | 0.248 |  |  |
| Current smoking | 0.97 (0.57-1.51) | 0.759 |  |  |
| Current drinking | 1.34 (0.82-2.18) | 0.241 |  |  |
| Left ventricular diameter | 1.03 (0.99-1.06) | 0.131 |  |  |
| Ejection fraction | 0.95 (0.90-1.00) | 0.055 |  |  |
| **History** | | | | |
| Hypertension | 2.35 (1.26-4.40) | 0.007 | 2.13 (1.23-4.04) | 0.020 |
| Diabetes | 1.10 (0.64-1.89) | 0.744 |  |  |
| Liver insufficiency | 2.40 (1.15-5.04) | 0.020 | 2.78 (1.32-5.85) | 0.007 |
| Chronic kidney disease | 2.17 (1.18-3.99) | 0.013 | 1.98 (1.07-3.66) | 0.031 |
| Previous MI | 2.06 (1.19-3.54) | 0.009 | 2.28 (1.32-3.94) | 0.003 |
| Previous coronary stent implantation | 1.11 (0.59-2.08) | 0.740 |  |  |
| Ischemic stroke | 1.55 (0.81-2.96) | 0.186 |  |  |
| Hyperlipemia | 0.68 (0.37-1.28) | 0.234 |  |  |
| Previous GI bleeding | 2.14 (0.78-5.87) | 0.142 |  |  |
| Hyperuricemia | 1.66 (0.72-3.84) | 0.237 |  |  |
| **Medication** |  |  |  |  |
| RAAS inhibitors | 1.81 (0.86-3.79) | 0.116 |  |  |
| Beta blockers | 0.84 (0.49-1.44) | 0.519 |  |  |
| Proton pump inhibitors | 1.28 (0.78-2.10) | 0.323 |  |  |
| Insulin | 1.47 (0.82-2.61) | 0.194 |  |  |
| Statins | 0.75 (0.23-2.38) | 0.621 |  |  |
| **Biomedical indicators** |  |  |  |  |
| Leucocyte | 1.06 (0.96-1.17) | 0.244 |  |  |
| Hemoglobin | 0.99 (0.98-1.00) | 0.030 |  |  |
| Platelets | 1.00 (0.99-1.00) | 0.149 |  |  |
| Mean platelet volume | 0.97 (0.85-1.12) | 0.690 |  |  |
| Platelet distribution width | 1.02 (0.93-1.11) | 0.672 |  |  |
| ALT | 1.01 (1.00-1.02) | 0.102 |  |  |
| eGFR | 0.98 (0.97-1.00) | 0.011 |  |  |
| Triglyceride | 0.91 (0.71-1.17) | 0.452 |  |  |
| Total cholesterol | 0.89 (0.71-1.13) | 0.340 |  |  |
| Hs-cTnI | 1.01 (0.97-1.06) | 0.595 |  |  |
| Glycosylated hemoglobin | 1.02 (0.87-1.19) | 0.838 |  |  |
| **Coronary arteriography** |  |  |  |  |
| Single-vessel disease | ref | 0.766 |  |  |
| Double-vessel disease | 0.81 (0.43-1.55) | 0.531 |  |  |
| Triple-vessel disease | 1.02 (0.58-1.77) | 0.956 |  |  |
| **Grouping** |  |  |  |  |
| ticagrelor vs clopidogrel (as reference) | 1.78 (1.04-3.03) | 0.035 |  |  |

95% CI, 95% confidence interval; HR, hazard ratio;

ACS, Acute coronary syndrome; ALT, Alanine aminotransferase; BMI, Body mass index; eGFR, Estimated glomerular filtration rate; GI, Gastrointestinal; Hs-cTnI: hypersensitive cardiac troponin I; LV, left ventricular; MI, Myocardial infarction; PCI, percutaneous coronary intervention; RAAS, Renin-angiotensin-aldosterone system.

**Supplemental table 4. Correlation between BARC with different antiplatelet drug regimens in ACS patients with LV dysfunction undergoing PCI.**

| **Variable** | **Univariable**  **HR (95% CI)** | **p value** | **Multivariable**  **HR (95% CI)** | **p value** |
| --- | --- | --- | --- | --- |
| Age, years | 1.03 (1.00-1.06) | 0.038 | 1.04 (1.01-1.07) | 0.018 |
| Gender (female vs male) | 0.96 (0.58-1.59) | 0.870 |  |  |
| BMI, kg/m^2^ | 1.00 (0.96-1.04) | 0.910 |  |  |
| Current smoking | 1.22 (0.76-1.96) | 0.403 |  |  |
| Current drinking | 0.77 (0.48-1.25) | 0.297 |  |  |
| Left ventricular diameter | 0.99 (0.96-1.03) | 0.753 |  |  |
| Ejection fraction | 0.99 (0.94-1.05) | 0.847 |  |  |
| **History** | | | | |
| Hypertension | 1.37 (0.81-2.32) | 0.245 |  |  |
| Diabetes | 0.95 (0.56-1.63) | 0.860 |  |  |
| Liver insufficiency | 1.88 (0.86-4.10) | 0.114 |  |  |
| Chronic kidney disease | 2.09 (1.16-3.75) | 0.014 | 1.97 (1.09-3.56) | 0.025 |
| Previous MI | 1.21 (0.67-2.17) | 0.531 |  |  |
| Previous coronary stent implantation | 1.71 (1.00-2.93) | 0.049 | 1.59 (0.92-2.73) | 0.098 |
| Ischemic stroke | 1.12 (0.56-2.25) | 0.755 |  |  |
| Hyperlipemia | 1.21 (0.71-2.05) | 0.479 |  |  |
| Previous GI bleeding | 2.22 (0.90-5.52) | 0.085 | 1.74 (0.64-4.74) | 0.276 |
| Hyperuricemia | 0.40 (0.10-1.63) | 0.200 |  |  |
| **Medication** |  |  |  |  |
| RAAS inhibitors | 0.79 (0.45-1.38) | 0.401 |  |  |
| Beta blockers | 0.89 (0.52-1.51) | 0.654 |  |  |
| Proton pump inhibitors | 1.44 (0.89-2.32) | 0.138 |  |  |
| Insulin | 1.06 (0.58-1.93) | 0.853 |  |  |
| Statins | 1.17 (0.29-4.76) | 0.829 |  |  |
| **Biomedical indicators** |  |  |  |  |
| Leucocyte | 1.00 (0.90-1.12) | 0.981 |  |  |
| Hemoglobin | 1.00 (0.99-1.01) | 0.342 |  |  |
| Platelets | 1.00 (1.00-1.00) | 0.889 |  |  |
| Mean platelet volume | 1.10 (0.96-1.00) | 0.183 |  |  |
| Platelet distribution width | 0.98 (0.89-1.05) | 0.600 |  |  |
| ALT | 1.00 (0.99-1.01) | 0.747 |  |  |
| eGFR | 1.00 (0.99-1.02) | 0.623 |  |  |
| Triglyceride | 1.18 (1.04-1.34) | 0.013 | 1.12 (0.97-1.28) | 0.116 |
| Total cholesterol | 0.96 (0.77-1.20) | 0.738 |  |  |
| Hs-cTnI | 1.01 (0.97-1.06) | 0.545 |  |  |
| Glycosylated hemoglobin | 1.17 (1.01-1.34) | 0.032 | 1.15 (0.99-1.33) | 0.062 |
| **Coronary arteriography** |  |  |  |  |
| Single-vessel disease | ref | 0.359 |  |  |
| Double-vessel disease | 0.73 (0.38-1.41) | 0.350 |  |  |
| Triple-vessel disease | 1.16 (0.69-1.97) | 0.579 |  |  |
| **Grouping** |  |  |  |  |
| ticagrelor vs clopidogrel (as reference) | 2.06 (1.22 -3.49) | 0.007 | 2.00 (1.17-3.40) | 0.011 |

95% CI, 95% confidence interval; HR, hazard ratio;

ACS, Acute coronary syndrome; ALT, Alanine aminotransferase; BMI, Body mass index; eGFR, Estimated glomerular filtration rate; GI, Gastrointestinal; Hs-cTnI: hypersensitive cardiac troponin I; LV, left ventricular; MI, Myocardial infarction; PCI, percutaneous coronary intervention; RAAS, Renin-angiotensin-aldosterone system.
